# Co-occurrence of *PML-RARA* gene fusion, chromosome 8 trisomy, and *FLT3* ITD mutation in a young female patient with de novo acute myeloid leukemia and early death: A CARE case report

**DOI:** 10.1097/MD.0000000000019730

**Published:** 2020-04-03

**Authors:** Florin Tripon, George Andrei Crauciuc, Alina Bogliş, Valeriu Moldovan, Johanna Sándor-Kéri, István Jr Benedek, Adrian Pavel Trifa, Claudia Bănescu

**Affiliations:** a Department of Medical Genetics; b Genetics Laboratory, Center for Advanced Medical and Pharmaceutical Research, George Emil Palade University of Medicine, Pharmacy, Science and Technology of TârguMureş; c Genetics Laboratory, Mures County Emergency Clinical Hospital (SCJU Târgu Mureş); d Department of Internal Medicine, George Emil Palade University of Medicine, Pharmacy, Science and Technology of TârguMureş, TârguMureş; e Department of Medical Genetics, University of Medicine and Pharmacy “Iuliu Haţieganu”, Cluj-Napoca, Romania.

**Keywords:** acute, chromosome 8, gene fusion, leukemia, myeloid, trisomy

## Abstract

**Rationale:**

Co-occurrence of cytogenetic and molecular abnormalities is frequently seen in patients with acute myeloid leukemia (AML). The clinical outcome and genetic abnormalities of AML may vary; therefore, genetic investigation must be complex, using several techniques, to have an appropriate characterization of the AML genome and its clinical impact. The available molecular markers can predict prognosis only partially. Acute promyelocytic leukemia subtype M3 **(**AML M3) is a subtype of AML characterized by the presence of promyelocytic leukemia-retinoic acid receptor alpha (*PML-RARA*) genes fusion. Targeted treatment with all-trans-retinoic acid (ATRA) and ATRA combined with arsenic trioxide significantly improved the survival of AML M3 patients. Unknown prognostic factors could contribute to the early death of these patients.

**Patient Concerns:**

We present the case of a young female (20 years old) patient, who presented at the emergency department 5 months after giving birth to her first child, complaining of asthenia, fatigue, general musculoskeletal pain, and fever (38°C), symptoms having been present for the previous 6 days. The patient denied any chronic diseases in her medical and family history.

**Diagnosis:**

Laboratory analysis revealed severe pancytopenia. Cytogenetic and molecular analyzes revealed chromosomal abnormalities (trisomy 8), *PML-RARA* gene fusion, and fms-like tyrosine kinase 3 *(FLT3)* gene mutation. The immunophenotypic analysis was also suggestive for AML M3 according to the FAB classification.

**Interventions:**

Specific treatment was initiated for AML M3 and for secondary conditions. Molecular and cytogenetic analyzes were performed to have a more detailed characterization of the patient's genome.

**Outcome:**

Seventy-two hours after admission, she developed psychomotor agitation, confusion, coma, and convulsion. Subsequent deterioration and early death were caused by intracerebral hemorrhage with multiple localization and diffuse cerebral edema.

**Lessons:**

The presence of *FLT3* internal tandem duplication (ITD) mutation may explain the rapid and progressive degradation of this AML M3 case and it may be used as a prognostic marker even when co-occuring with other markers such as *PML-RARA* gene fusion and trisomy 8. We consider that *FLT3* ITD mutation analysis in young patients with AML should be performed as soon as possible. New strategies for patients’ education, AML (or cancers in general) prevention, and treatment are needed.

## 1 Introduction

Acute myeloid leukemia (AML) is a heterogeneous disease characterized by various cytogenetic and molecular abnormalities.^[1–3]^ Recent advances in molecular genetics provide new molecular markers for AML diagnosis, risk stratification, prognosis, or treatment by using next generation sequencing (NGS) gene panels. Molecular markers, such as fms-like tyrosine kinase 3 *(FLT3)*, nucleophosmin 1 (*NPM1*), CCAAT/enhancer-binding protein alpha (*CEBPA*), runt-related transcription factor 1 (*RUNX1*), additional sex combs like 1, and tumor protein p53 (*TP53)* genes, were introduced in the latest European LeukemiaNet (ELN) recommendations.^[4]^ Co-occurrence of cytogenetic and molecular abnormalities are frequently present in AML patients. Moreover, the clinical outcome and genetic abnormalities are different according to the age of the patients.^[5]^ Therefore, the genetic investigations of patients with AML should be complex, using various techniques, to have an appropriate characterization of the AML genome and its clinical impact, because the available molecular markers can only partially predict prognosis for AML.^[6]^ Acute promyelocytic leukemia subtype M3 (AML M3) is a subtype of AML characterized by the presence of promyelocytic leukemia-retinoic acid receptor alpha (*PML-RARA)* genes fusion.^[7,8]^ Targeted treatment with all-trans retinoic acid (ATRA) and ATRA combined with arsenic trioxide significantly improved the survival of AML M3 patients. However, unknown prognostic factors could contribute to the early death of these patients.^[8]^

Here, we describe the clinical outcome of a young AML M3 female patient, with chromosome 8 trisomy, *PML-RARA* gene fusion, and *FLT3* internal tandem duplication (ITD) mutation, who was diagnosed with AML 5 months after she gave birth to her first child. Genetic investigations included conventional cytogenetic analysis and molecular techniques, such as ligation-dependent reverse transcription polymerase chain reaction (LD-RT PCR), multiplex ligation–dependent probe amplification (MLPA), and NGS.

## 2 Methods

The Ethics Committee of the Clinical and Emergency County Hospital from TârguMureş, Romania, approved this study (No. 10665/2019). The patient has provided informed consent for publication of the case and signed the informed written consent for genetic testing.

### 2.1 Immunophenotyping analysis

Immunophenotyping analysis was made from bone marrow aspirate.

### 2.2 Genetic analysis

Conventional cytogenetic analysis was performed from bone marrow obtained by biopsy and peripheral leucocytes according to protocols previously described.^[9,10]^ In parallel, we initiated molecular analysis for *FLT3* (ITD and D835), *NPM1* c.863_864ins, and *DNMT3A* R882 mutations by using different polymerase chain reaction (PCR) methods (PCR, amplification refractory mutation system polymerase chain reaction, restriction fragment length polymorphism, and fragment analysis), as previously reported.^[9]^ In the second day, we performed MLPA analysis using 3 different kits (SALSA MLPA P036, P070, P377, MRC-HOLLAND) and LD-RT PCR to detect 57 specific acute leukemia gene fusions according to the protocol previously described (capillary sequencing was performed to sequence the amplicon).^[11–13]^ NGS was carried out using a community panel, Ion AmpliSeq AML Research Panel, and Ion Proton system (Thermo Fisher Scientific USA).

### 2.3 Case presentation

The patient, a young female (20 years old), presented to the Emergency Department complaining of asthenia, fatigue, general musculoskeletal pain, and fever (38°C), symptoms having been present for the previous 6 days. The patient denied any chronic diseases in her medical and family history. Laboratory analysis revealed severe pancytopenia. The patient was immediately admitted to the hematology department for further investigations and treatment. Before initiating any treatment, bone marrow aspiration was performed and sent for immunophenotypic, conventional cytogenetic, and molecular analyzes. The immunophenotypic analysis revealed a percentage of 86% myeloid elements positive for CD45, CD33, CD13, CD11b+ (4.1%), CD11c (10%), CD64 (81%), CD34 (5%), CD117 (15%), CD123, CD38 (30%), CD4 (8%), CD22 (9.5%), ic MPO and negative for CD15, CD14, CD16, CD36, HLA-DR, CD3, CD5, CD7, CD19, CD10, ic CD3, and ic CD79a. The results were suggestive for AML M3 according to the FAB classification. Therefore, the patient received treatment with ATRA and idarubicin. Laboratory analysis (mentioning only abnormal values) revealed high values of white blood cells count, MR-pro-atrial natriuretic peptide, and ultra sensitive C-reactive protein and low values of red blood cells and platelets. Blood culture analysis showed positive results for *Methicillin-sensitive Staphylococcus aureus* (MSSA) and targeted treatment was initiated. In the following hours, the patient presented disseminated intravascular coagulation and received treatment consisted of pooled platelets and red blood cells, fresh blood plasma, and hemostatic agents.

**Figure 1:**
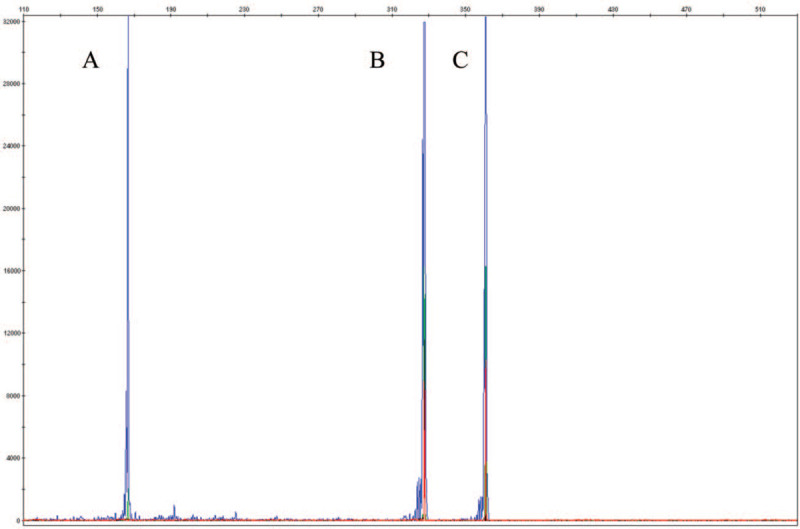
The multiplex fragment analysis of nucleophosmin 1 (*NPM1*) and fms-like tyrosine kinase 3 (*FLT3*) internal tandem duplication (ITD) mutations. A, The peak for *NPM1* wild type allele (166 bp and 32377 height). B, The peak for *FLT3* ITD wild type allele (327 bp and 31,939 height). C, The peak for *FLT3* ITD mutation [360 bp- 11 trinucleotide repetition and 32,277 height- variant allele frequency (VAF) 50%].

**Figure 2:**
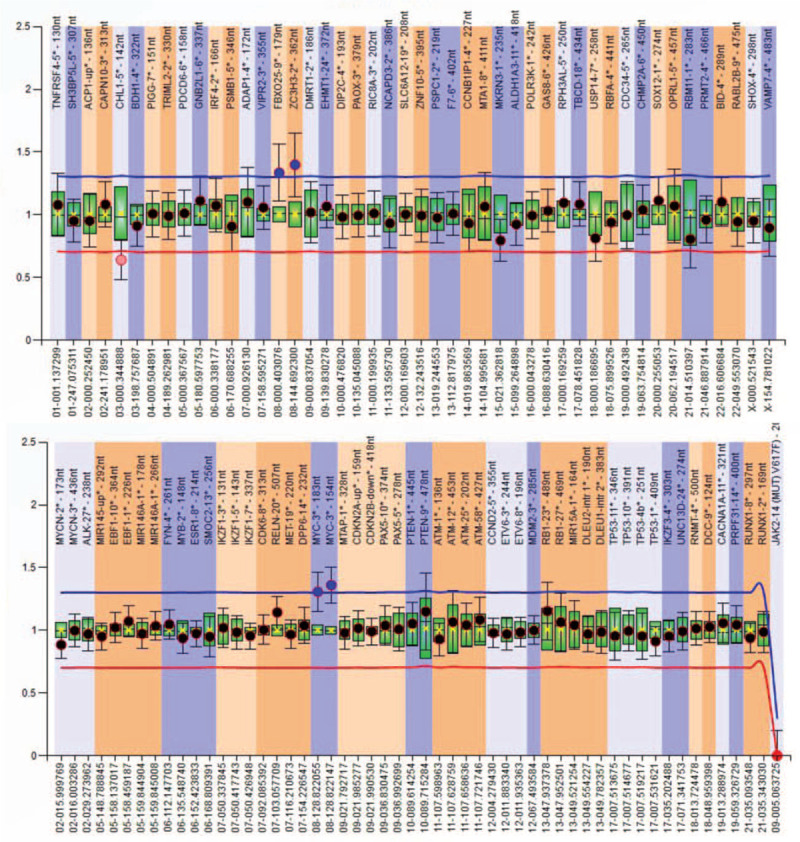
Multiplex ligation-dependent probe amplification (MLPA) results. A, MLPA P036 probemix (up). B, MLPA P070 probemix (down).

**Figure 3:**
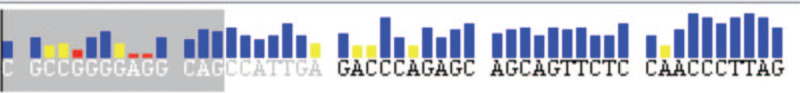
The sequence of promyelocytic leukemia-retinoic acid receptor alpha (*PML-RARA*) gene fusion. Marked with grey color is the sequence specific for *PML* gene followed by a sequence specific for *RARA* gene.

Molecular analysis by PCR technique detected the presence of the *FLT3* ITD mutation. For confirmation and quantification of the mutant clone, fragment analysis was performed. Figure 1 illustrates the fragment analysis results with 50% variant allele frequency (VAF). MLPA analysis showed duplication signal for 8q and 8p chromosome, suggesting of trisomy 8 (Fig. 2). No other mutations or copy number changes (CNCs) were found. LD-RT PCR analysis confirmed the presence of PML-RARA fusion (Fig. 3).

After short-term culture of bone marrow cells, the conventional cytogenetic analysis confirmed chromosome 8 trisomy. NGS analysis was performed to investigate genes such as *CEBPA*, *RUNX1*, *ASXL*, *TP53*, and other 15 genes, which might be difficult to analyze with other molecular techniques. Five missense variants were identified by NGS analysis, namely rs2276599 (*DNMT3A* c.1122+7G>A, homozygous with the variant allele), rs17253672 (*TET2* c.1088C>T, heterozygous genotype), rs34402524 (*TET2* c.5162T>G, heterozygous genotype), rs2454206 (*TET2* c.5284A>G, heterozygous genotype), and rs1042522 (*TP53* c.215C>G, heterozygous genotype, variant allele ratio 0.153). According to VarSome and ClinVar, the *TP53* c.215C>G variant has an uncertain significance, and the other identified variants are considered benign.

After 72 hours, the molecular results (somatic mutations, MLPA, and LD-RT PCR) were communicated to the hematology department. At the same time, the patient developed psychomotor agitation, confusion, coma, and convulsion. Specific treatment was administrated, and brain computed tomography was performed. Intracerebral hemorrhage with multiple localization and diffuse cerebral edema were the cause of her deterioration. The patient was transferred to the neurology department due to her poor medical status, but unfortunately, she died within 24 hours.

## 3 Discussion

Our investigated young patient with AML presented chromosome 8 trisomy, *PML-RARA* gene fusion and *FLT3* ITD mutations (VAF = 50%) simultaneously.

Although chromosome 8 trisomy is the most frequent genetic aberration found in AML patients,^[14]^ its prognostic significance is not completely cleared. Moreover, one study concludes that trisomy 8 cannot be considered as a prognostic marker,^[15]^ since this chromosomal aberration co-occurs with other genetic alterations such as monosomy 5, monosomy 7, several gene fusions, or gene mutations. One previous study suggests that prognosis of patients with AML with trisomy 8 is determined by the accompanying genetic alterations (if any exist).^[14]^ Another study suggests that trisomy 8 is an intermediate prognostic factor in patients with AML and a poor prognosis factor for chronic myeloid leukemia (being associated with blastic phase).^[16]^

The fact that chromosome 8 trisomy usually coexists with other genetic alterations, is currently accepted. Trisomy 8 is considered to be a disease-modulating secondary event, that may associate cryptic translocations, deletions (del), or gene mutations as the most important primary events.^[16]^ Chromosome 8 trisomy influences the expression of genes localized on chromosome 8, alters global gene expression,^[17]^ and seems to have various consequences in the different AML subtypes.^[18]^ Therefore, the genetic investigation of patients with AML with trisomy 8 (and not only) should be complex using various techniques to overcome the limitation of each of them.

In most cases, the *PML-RARA* fusion has a favorable prognosis even in the presence of a secondary abnormality such as trisomy 8.^[19,20]^*FLT3* ITD mutation is considered an unfavorable marker associated with short overall survival and disease-free survival, as well as with high risk for AML relapse.^[3,21–23]^ According to ELN 2017, patients with AML with allelic ratio greater than 0.5 for *FLT3* ITD mutation and negative for *NPM1* mutation are considered to have an adverse risk status.^[4,24]^ The presence of *FLT3* ITD mutation in our patient could explain the rapid and progressive degradation, and it could be used as an adverse prognostic marker even if it co-occurs with favorable prognostic markers such as *PML-RARA* fusion and chromosome 8 trisomy. Frequent co-occurrence of trisomy 8 and *FLT3* ITD mutation in young AML patients has been previously described.^[25]^ Therefore we consider that *FLT3* ITD mutation analysis in young patients with AML should be performed as soon as possible. In our laboratory molecular genetic tests (somatic mutations, MLPA, and LD-RT PCR) were completed in 72 hours, but for patients with AML, we should be able to detect mutations more quickly and more efficiently, thus improving the outcome and health education of these patients. Also, there may be insufficient awareness of leukemia and of cancer in general among young individuals. Previous studies on female breast cancer conclude that the occupation and education level of the patients are independent factors for tumor staging; therefore, new strategies, publicity, and education programs are needed focusing on cancer prevention and treatment.^[26,27]^

Moreover, one study investigating the causes of early death in AML M3 patients, concludes that “the current therapeutic strategies to reduce the incidence of early death in these cases are not adequate and will benefit from focused research attention.”^[28]^ Therefore, it is recommended for patients who present nonspecific symptoms for prolonged periods, to consult a physician as soon as possible for investigations. One possible solution may be represented by personalized treatment, like FLT3 tyrosine kinase inhibitors in adult patients with AML with *FLT3* ITD mutation.

Regarding molecular techniques used for patient investigations, we admit that MLPA is a fast multiplex technique, useful, and cost-effective^[29,30]^ which could be performed for rapid identification of aneuploidy in patients with leukemia by using subtelomeric (P036 and P070) or centromeric (P181 or P182) MLPA probe mixes as previously described by Vázquez-Reyes et al.^[31]^ As we previously reported,^[32]^ MLPA is an efficient technique for AML patient's investigation, the presence of CNCs in patients with Eastern Cooperative Oncology Group performance status ≥3 being correlated with higher risk for death.^[32]^ LD-RT PCR is a promising low-cost (approximately 15 euro/patient) and rapid (final results in 2 days) multiplex technique, which may be used for the investigation of gene fusions in patients with AML^[12]^ and not only.^[12,13]^ We consider that fragment analysis is the criterion standard for *FLT3* (ITD and D835) and *NPM1* mutation investigation, being able to predict a more appropriate outcome.^[33]^

In conclusion, we consider that *FLT3* ITD mutation with a high VAF, associated with *PML-RARA* gene fusion and chromosome 8 trisomy represents a poor prognostic for AML and in young patients may lead to early death. The genetic investigation of patients with AML with trisomy 8 (and not only) must be complex, to overcome the limits of each technique, to have an appropriate characterization of the genome, a realistic prognosis, and personalized treatment. New strategies for patients’ education, AML prevention, and treatment are needed.

## Author contributions

**Conceptualization:** Florin Tripon, Johanna Sándor-Kéri, Claudia Banescu.

**Data curation:** Florin Tripon, Alina Boglis, George Andrei Crauciuc, István Jr Benedek.

**Formal analysis:** Florin Tripon, Alina Boglis, George Andrei Crauciuc, István Jr Benedek.

**Funding acquisition:** Florin Tripon.

**Investigation:** Valeriu Moldovan, Johanna Sándor-Kéri, István Jr Benedek, Claudia Banescu.

**Methodology:** István Jr Benedek, Claudia Banescu.

**Project administration:** Florin Tripon.

**Resources:** Florin Tripon.

**Software:** Alina Boglis, George Andrei Crauciuc.

**Supervision:** Claudia Banescu.

**Validation:** Adrian Pavel Trifa, Claudia Banescu.

**Visualization:** Adrian Pavel Trifa.

**Writing – original draft:** Florin Tripon.

**Writing – review & editing:** Valeriu Moldovan, Adrian Pavel Trifa, Claudia Banescu.

Florin Tripon orcid: 0000-0002-4297-9988.
